# An evaluation of the efficacy and the safety of home blood pressure monitoring in the control of hypertensive disorders of pregnancy in both pre and postpartum periods: a systematic review and meta-analysis

**DOI:** 10.1186/s12884-023-05663-w

**Published:** 2023-08-01

**Authors:** Muayad Albadrani, Muhammad Tobaiqi, Sami Al-Dubai

**Affiliations:** 1 Department of Family and Community Medicine, College of Medicine, Taibah University, Al-Madinah Al-Munawwarah, Saudi Arabia; 2 Joint Program of Saudi Board of Preventive Medicine Madinah, Madinah Health Cluster, Al-Madinah, Saudi Arabia

**Keywords:** Home blood pressure monitoring, Hypertensive disorders of pregnancy, Meta-analysis, Systematic review

## Abstract

**Background:**

Hypertensive disorders of pregnancy (HDP) can significantly impact maternal, neonatal, and fetal health. For controlling these disorders, frequent blood pressure measurements are required. Home blood pressure monitoring (HBPM) is a suggested alternative to conventional office monitoring that requires frequent visits. This systematic review was conducted to evaluate the efficacy and safety of HBPM in the control of HDP.

**Methods:**

We systematically conducted databases search for relevant studies in June 2022. The relevant studies were identified, and qualitative synthesis was performed. An inverse variance quantitative synthesis was conducted using RevMan software. Continuous outcome data were pooled as means differences, whereas dichotomous ones were summarized as risk ratios. The 95% confidence interval was the measure of variance.

**Results:**

Fifteen studies were included in our review (*n* = 5335). Our analysis revealed a superiority of HBPM in reducing the risk of induction of labor, and postpartum readmission (*P* = 0.02, and 0.01 respectively). Moreover, the comparison of birth weights showed a significant variation in favor of HBPM (*P* = 0.02). In the analysis of other outcomes, HBPM was equally effective as office monitoring. Furthermore, HBPM did not result in an elevated risk of maternal, neonatal, and fetal adverse outcomes.

**Conclusion:**

Home monitoring of blood pressure showed superiority over office monitoring in some outcomes and equal efficacy in other outcomes.

**Supplementary Information:**

The online version contains supplementary material available at 10.1186/s12884-023-05663-w.

## Introduction

Hypertensive disorders of pregnancy (HDP) affect around 10% of pregnancies globally, with preeclampsia accounting for 4% and gestational hypertension (without proteinuria) accounting for 6% [1, 2]. These disorders were identified to affect maternal, neonatal, and fetal health resulting in significant morbidity and mortality [1]. As reported in the US, HDP constituted a major cause of postpartum obstetrical readmission; indicating the persistence and progression of the disease [3]. Moreover, HDP was shown to have a long-term impact on women's cardiovascular health [4, 5]. These women have a greater risk of developing renal dysfunction, stroke, and persistent chronic hypertension, among other cardiovascular diseases [6–8]. Therefore, effective and sustainable monitoring and management of hypertension should be implemented to prevent such incidents. Traditionally, blood pressure is monitored in health centers, necessitating frequent office visits which might be inconvenient for most women. However, the need for blood pressure monitoring should not be ignored.

Home blood pressure monitoring (HBPM) is a promising alternative to in-office monitoring that is recommended by international guidelines [9]. Monitoring blood pressure at home has frequently shown a convenient and effective blood pressure control among nonpregnant hypertensive adults [10–12]. Recently, it was suggested that HBPM could replace frequent office visits for screening HDP [13]. The implementation of HBPM protocols can reduce the number of required office visits that constitute a financial burden on pregnant women and the health system as well [14]. In addition, HBPM is a convenient alternative to office visits that may guarantee better compliance [13]. More importantly, HBPM is more efficient in detecting the alterations in blood pressure that occur between office visits, as well as reducing white-coat hypertension risk [14, 15].

Several clinical trials have compared blood pressure monitoring at home to office visits in controlling HDP, however, their results have shown some heterogeneity. We conducted this systematic review with a meta-analysis to reach conclusive evidence on HBPM efficacy and safety in the control of HDP.

## Methods

We followed the guidance of the Cochrane handbook for systematic reviews of intervention in conducting this study [16]. Thereafter, we reported our manuscript in accordance with the Preferred Reporting Items for Systematic reviews and Meta-Analysis (PRISMA) [17].

### Search strategy and information source

In June 2022, we conducted a systematic databases search using the following search strategy: (“Ambulatory Blood Pressure Monitoring” OR “Monitoring, Ambulatory Blood Pressure” OR “Blood Pressure Monitoring, Self” OR “Self Blood Pressure Monitoring” OR “Blood Pressure Monitoring, Home” OR “Home Blood Pressure Monitoring”) AND (“Hypertension*, Pregnancy Induced” OR “Pregnancy-Induced Hypertension” OR “Pregnancy Induced Hypertension” OR “Induced Hypertension*, Pregnancy” OR “Gestational Hypertension” OR “Hypertension, Gestational” OR “Transient Hypertension, Pregnancy” OR “Hypertension, Pregnancy Transient” OR “Pregnancy Transient Hypertension”).

We conducted our search on PubMed, Scopus, Web of Science, and Cochrane library from their inception for any relevant results. Following this, a manual search was conducted in the reference lists of the identified relevant articles.

### Eligibility criteria and studies selection

This review included systematically the studies that investigated the use of HBPM in comparison with conventional office monitoring in the control of HDP or normal pregnant women at high risk of HDP. Studies enrolling pregnant or postpartum women with established HDP or normal pregnant women at high risk of HDP were eligible for inclusion in our review. For the studies to be eligible for this systematic review, the efficacy, and safety of HBPM should be investigated. Studies enrolling women younger than 18, having inaccessible full texts, or cost-effectiveness studies were not eligible for inclusion in this review.

Following the removal of the duplicates, eligible studies were selected through two-step screening. Initially, the titles in addition to the abstracts of the retrieved search results were reviewed for any relevant study. After that, the full texts of the identified relevant studies were screened carefully for final eligibility.

### Quality assessment

Cochrane collaboration tool for risk of bias assessment tool was used to evaluate the quality of the evidence provided in the eligible randomized controlled trials (RCTs) [18]. For non-randomized clinical trials, the RoBANS tool of Cochrane collaboration (Risk of Bias Assessment tool for Non-randomized Studies) was used to determine the quality [19]. Furthermore, the quality of cohort and case–control studies was evaluated by the quality assessment tool provided by the National Institute of Health [20].

### Study measures

Data that summarize the included studies’ key features were extracted in a table, these data included the site and design of the study, eligibility criteria for the participants, type of HBPM device used, follow-up duration, and the study outcomes. In addition, the baseline characteristics of the enrolled women in each study were summarized. These baseline data included the women's age, race, body mass index (BMI), percentage of nulliparous women, and the weeks of gestation at study entry. Concerning the investigated studies' outcomes, both maternal and neonatal outcomes were studied. The studied efficacy and safety outcomes included the risk of preeclampsia, induction of labor, caesarian delivery, and postpartum readmission. Moreover, the percentages of live births and preterm deliveries were analyzed with the gestational age at delivery. Furthermore, we studied the birth weight in addition to the risk of intrauterine growth restriction, delivering a neonate who is small for gestational age, and admission to the neonatal intensive care unit (NICU). The adverse maternal, neonatal, and fetal outcomes were studied as well.

### Data synthesis

The statistical analysis of maternal and neonatal outcomes in this meta-analysis was performed using RevMan software (v 5.3) in an inverse variance method. The statistical pooling of continuous efficacy outcome data was conducted in the form of mean difference (MD), whereas all the categorical dichotomous data were pooled in the form of risk ratio (RR). The variance measure was the 95% confidence interval (CI). Visual assessment of the forest plot, in addition to I-square (I2) and chi-square tests were used for the evaluation of heterogeneity among the included studies' results. Heterogeneity was considered statistically significant when the I2 value is ≥ 50%, here, a random-effect analysis model was used rather than the fixed-effect model [21]. According to the Cochrane handbook, one or two studies with inconsistent findings may cause heterogeneity. Excluding studies from meta-analyses based on their results may cause bias. If the outlying result has an obvious cause, the study may be dismissed with more confidence. This criterion is unreliable since every study in a meta-analysis has at least one distinguishing feature. For this reason, we left one study out when the results were heterogeneous to solve this heterogeneity and we did sensitivity analyses with and without outliers [21, 22].

## Results

### Studies selection and characteristics

**Fig. 1:**
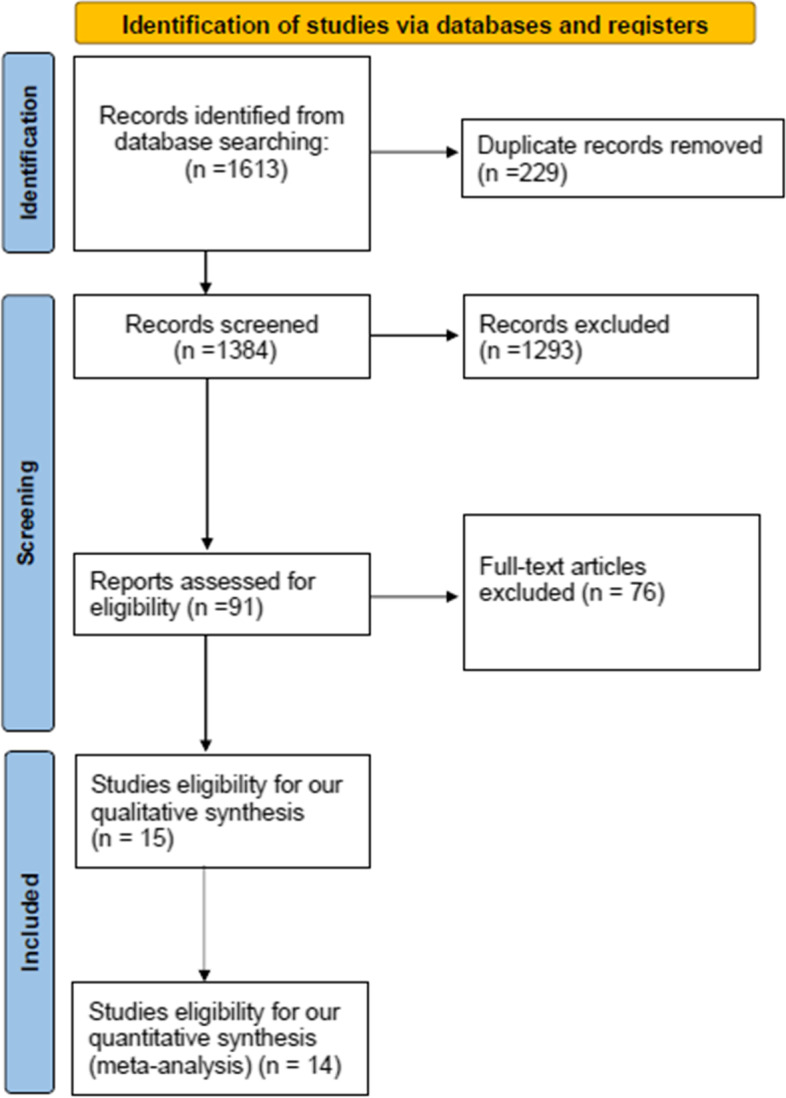
The PRISMA flow chart

Our predetermined systematic search retrieved 1613 results, among which 229 were duplicated. Following the removal of the duplicated results, 1384 were eligible for the title and abstract screening. With the titles and abstracts screened, 1293 studies were excluded and only 91 were eligible for the full-text screening. Finally, 15 studies were included in our systematic review [13, 23–36]. Among those, 14 studies were included in the quantitative synthesis [13, 23–25, 27–36] (Fig. 1). The primary features of the eligible studies are described in Table 1. An overall number of 5335 women were enrolled, their baseline characteristics are summarized in Table 2.

**Table 1:** Summary of the included studies

Study ID	Site	Study Design	Inclusion criteria	HBPM device	Primary outcome	Auxiliary outcomes	Duration of Monitoring/follow up	Conclusion
**Cairns et al. 2018** [28]	UK	RCT	1. Women over the age of 18 with gestational hypertension or preeclampsia (as defined by the National Institute of Health and Care Excellence) 2. Who needed antihypertensive medication were eligible	Automated microlife	1. Feasibility	Mean BP measurements, mean arterial pressure, postnatal admission rates, side effects, quality of life scores	6 months	“This was the first randomized trial of postpartum blood pressure self-management. Self-management resulted in superior diastolic blood pressure control for up to 6 months.”
**Chappell et al. 2022** [36]	UK	RCT	1. Individuals aged 18 years or older were eligible if they had chronic hypertension (defined as sustained systolic BP ≥ 140 mmHg and/or diastolic BP ≥ 90 mmHg 2. Present at booking or before 20 weeks’ gestation3. OR receiving antihypertensive treatment outside pregnancy or at the time of referral) and were recruited up to 37 weeks gestation, or gestational hypertension (defined as sustained systolic BP ≥ 140 mmHg and/or diastolic 1 BP ≥ 90 mmHg after 20 weeks’ gestation), recruited at 20 to 37 weeks’ gestation	Automated Microlife ‘WatchBP Home’	1. Difference in mean systolic BP	Maternal and perinatal outcomes	Till delivery and until 8 weeks after birth	“Among pregnant individuals with chronic or gestational hypertension, blood pressure self-monitoring with telemonitoring compared with usual care did not lead to significantly improved clinic-based blood pressure control”
**Denolle et al. 2008** [35]	France	RCT	1. Patients were eligible if they had newly diagnosed hypertension, defined as the mean of three office BP readings taken at a single visit of 140/90 and 180/105 mm Hg after 18 weeks gestation	OMRON 705C	1. Prevalence and prognosis of white coat hypertension	Feasibility, safety, and cost savings of HBPM	7 days	“White Coat Hypertension was a relatively common and harmless condition. HBP monitoring was a viable and well-recognized option. Teletransmission, on the other hand, was required for safety.”
**Fukushima et al. 2002** [30]	USA	Cohort	1. Patients in this study had a blood pressure of 160/110 mm Hg 2. Were checked at least three times 3. Delivered at least eight days after the first test 4. Were outpatients for at least four days 5. Were at least 20 weeks pregnant	Accutor 3 (Datascope Corp., Paramus, NJ)	1. Delivery outcomes	Non	2 weeks	“Adjunctive cardiovascular dynamics monitoring may be useful in assessing and managing hypertension during pregnancy.”
**Hirshberg et al. 2018** [27]	USA	RCT	1. Women had to be above the age of 18 2. Who be able to speak and understand English 3. Who have access to a smartphone with unlimited text messaging capabilities	Not specified	1. Percentage of patients whose single BP was obtained in the first days of discharge 2. Percentage of patients in whom BP values were obtained at 72 h and 7–10 days	Initiation of medication, number of additional postpartum visits, patient satisfaction, and future health-awareness	2 weeks	“Text-based monitoring was more successful than conventional office-based follow-up in acquiring blood pressure and satisfying current clinical recommendations in women with pregnancy-related hypertension in the immediate post-discharge interval.”
**Holm et al. 2019** [26]	Denmark	RCT	1. At gestational week 12, pregnant women got regular ultrasounds	A&D TM-2655 BP Kiosk station	1. Mean arterial pressure	Measurement errors	NR	“There was no significant change in mean arterial pressure (MAP) between the two approaches. Erroneous blood pressure measures should be detected and repeated.”
**Hoppe et al. 2020** [29]	USA	Non-RCT	1. Participants with Hypertensive disorders during pregnancy	Bluetooth BP monitor	1. Hypertension related readmission	Non	6 weeks	“Telehealth with remote blood pressure monitoring and regular postpartum hypertension management reduced readmissions. Telehealth using remote blood pressure monitoring may help increase blood pressure collection, detect and treat uncontrolled hypertension, and reduce hospital readmissions.”
**Kalafat et al. 2019** [31]	UK	Case–control	1. Subjects with gestational diabetes were diagnosed using the International Society for the Study of Hypertension in Pregnancy (ISSHP) criteria	Automated Microlife ‘WatchBP Home’	Not stated	Adverse fetal, neonatal and maternal outcomes and number of antenatal hospital visits	4.8 weeks (1.8 – 8.6)	“HBPM reduces prenatal visits for women with GH compared to usual treatment. Fetal, neonatal, and mother outcomes were similar. Rare unfavorable pregnancy outcomes need large multicenter trials.”
**Lanssens et al. 2018–1** [32]	Belgium	Cohort	1. Participants with gestational hypertensive disorders	CE-approved device	1. Prenatal follow-up	Delivery outcomes	NR	“This study proved that remote monitoring allows for the provision of timely interventions to pregnant women in need.”
**Kitt et al. 2021** [23]	UK	RCT	1. Women over the age of 18 with gestational hypertension or preeclampsia (as defined by the National Institute of Health and Care Excellence) 2. Who needed antihypertensive medication were eligible	Automated microlife	1. Blood pressure values	Non	6 months	“Interventions to enhance BP management during the puerperium in women with hypertensive pregnancies improved BP in the long run, in a group at high risk of developing chronic hypertension and significant adverse cardiovascular events.”
**Pealing et al. 2019** [25]	UK	RCT	1. Women over the age of 18 with a singleton pregnancy with chronic or gestational hypertension but no preeclampsia	Automated microlife	1. Feasibility	Maternal and perinatal outcomes	3 months	“When compared with clinic monitoring, BP self-monitoring for the treatment of hypertension during pregnancy is possible and well-tolerated by women.”
**Perry et al. 2018** [13]	UK	Case–control	1. Pregnant women with chronic hypertension, gestational hypertension, or a high risk of pre-eclampsia, 2. No considerable proteinuria (≤ 1 + proteinuria on dipstick tests) 3. Normal biochemical and hematological indicators	Automated Microlife ‘WatchBP Home’	1. Number of visits to antenatal services	Adverse maternal and fetal outcome	8.9 weeks (3.4–16.5)	“In hypertensive pregnancies, HBPM has the potential to minimize the number of hospital visits required by patients while maintaining maternal and pregnancy outcomes.”
**Rayburn et al. 1985** [33]	USA	Case–control	1. Women who qualified for enrolment had documented pre-existing hypertension prior to being seen initially in the first or early second trimester	Not specified	1. Antenatal and perinatal outcomes	Non	24 weeks (8–35)	“In comparison to prior experience without home blood pressure monitoring, awareness of daily blood pressure variations outside the clinic led to fewer antepartum hypotension and the prescription of fewer antihypertensive drugs.”
**Rhodes et al. 2017** [34]	UK	Feasibility trial	1. Pregnant women with untreated diastolic blood pressure values of 90 mmHg or higher were eligible for the experiment 2. The average of three CBP measurements, taken 1 min apart, was used to diagnose hypertension	semi-automatic device	1. Feasibility	Adverse outcomes	6 weeks	“HBPM was feasible and acceptable to pregnant women.”
**Tucker et al. 2022** [24]	UK	RCT	1. Pregnant women aged 16 to 24 weeks of pregnancy with a greater risk of preeclampsia	Automated microlife	1. Time from randomization to the first recording of “clinic hypertension”	Maternal and perinatal outcomes	Till delivery	“Blood pressure self-monitoring with telemonitoring did not result in substantially earlier clinic-based identification of hypertension among pregnant women at high risk of preeclampsia as compared to conventional treatment.”

*Abbreviations*: *HBPM* Home blood pressure management, *NR* Not reported, *ACOG* American College of Obstetricians and Gynecologists, and *BP* Blood pressure

**Table 2:** Baseline characteristics of the enrolled patients in the included studies

Study ID	Study arms	Sample	Age, years, M ± SD	BMI, kg/m2, M ± SD	Nulliparous	Median gestation at entry, wk (IQR)	Race, white/black/others
**Cairns et al. 2018** [28]	HBPM	45	31.7 ± 5.3	29 ± 7.5	32 (71%)	35.9 (31.9–37.7)	41/4
	Control	46	31.7 ± 5.3	28 ± 8.3	31 (67%)	34.7 (31.7–36.9)	43/3
**Chappell et al. 2022** [36]	Chronic Hypertension (HBPM)	233	36 ± 5.4	30.7 (26.7–34.7)^a^	85 (36.5%)	18.6 (15.3–23.3)	115/70/38
	Chronic Hypertension (Control)	221	35.5 ± 5.8	30.5 (26.3–35.8)^a^	77 (34.8%)	18.3 (15.4–23.3)	109/71/41
	Gestational Hypertension (HBPM)	197	33.5 ± 6.1	29.4 (24.8–35.1)^a^	103 (52.3%)	34.3 (29.7–35.9)	141/17/39
	Gestational Hypertension (Control)	199	33.6 ± 5.6	28.5 (25–35.4)^a^	101 (50.8%)	33.9 (30.3–36.1)	137/22/40
**Denolle et al. 2008** [35]	HBPM	24	27 ± 3	-	39 (82%)	29 ± 5$	-
	Control	24		-			-
**Fukushima et al. 2002** [30]	HBPM	19	29.7 ± 7.6	-	71 (36%)	19.6 ± 9.8$	-
	Control	180		-		27.5 ± 9.3$	-
**Hirshberg et al. 2018** [27]	HBPM	103	28 ± 6	30.1 (24.3–33.8)^a^	61 (59.2%)	38 (36–39)	28/68/7
	Control	103	28 ± 5	31.0 (25.1–38.3)^a^	52 (50.5%)	38 (36–39)	25/73/5
**Holm et al. 2019**	HBPM	80	-	-	-	-	-
	Control		-	-	-	-	-
**Hoppe et al. 2020** [29]	HBPM	214	-	-	-	-	-
	Control	214	-	-	-	-	-
**Kalafat et al. 2019** [31]	HBPM	80	34.0 (30.0–37.0)^a^	26.4 (23.6–30.0)^a^	59 (73.7%)	34.0 (28.2–36.3)	-
	Control	63	31.0 (28.0- 33.5)^a^	27.1 (24.2–30.3)^a^	51 (80.9%)	36.0 (33.0–37.3)	-
**Lanssens et al. 2018–1** [32]	HBPM	86	30.97 ± 5.61	26.79 ± 5.36	-	10.51 ± 6.11$	-
	Control	215	30.53 ± 5.17	28.38 ± 6.67	-	10.60 ± 5.52$	-
**Kitt et al. 2021** [23]	HBPM	30	35.2 ± 5.3	28.5 (25.6–33.3)^a^	0	-	-
	Control	31	34.1 ± 5.3	27.7 (23.9–31.4)^a^	0	-	-
**Pealing et al. 2019-Chronic Hypertension** [25]	HBPM	55	35.9 ± 5.6	31 ± 7	17 (31%)	16.6 (12.9–20.1)	27/21/7
	Control	31	31.7 ± 5.3	31.9 ± 7	9 (29%)	14.9 (13.0–20.0)	21/8/3
**Pealing et al. 2019-Gestational Hypertension** [25]	HBPM	49	33.4 ± 5.9	29.5 ± 7.1	20 (41%)	35.0 (32.4–36.1)	37/11/1
	Control	23	34.2 ± 5.1	27.6 ± 6.4	14 (61%)	34.7 (32.1–36.4)	18/4/1
**Perry et al. 2018** [13]	HBPM	108	32.5 (29.0–37.8)^a^	27.7 (23.8–33.2)^a^	61 (56.5%)	30.0 (22.0–35.0)	-
	Control	58	32.0 (28.0–35.3)^a^	27.9 (24.9–31.2)^a^	32 (55.2%)	33.6 (28.2–36.1)	-
**Rayburn et al. 1985** [33]	HBPM	33	31 ± 3	11 (33.3%)	-	35 ± 3$	-
	Control	34	30 ± 4	14 (41.2%)	-	36 ± 3$	-
**Rhodes et al. 2017** [34]	HBPM	51	26.2 ± 4.3	32 (62%)	-	-	-
	Control	49	27.3 ± 6	32 (65%)	-	-	-
**Tucker et al. 2022** [24]	HBPM	1220	32.8 ± 5.7	26.5 (22.7–32.1)^a^	745 (61.1%)	-	887/88/236
	Control	1217	33 ± 5.6	26.1 (22.6–32.4)^a^	742 (61.0%)	-	914/99/204

*Abbreviations*: *HBPM* Home blood pressure management, *BMI* Body mass index. ^a^data represented as median and IQR, $: data represented as M ± SD

### Quality assessment

**Fig. 2:**
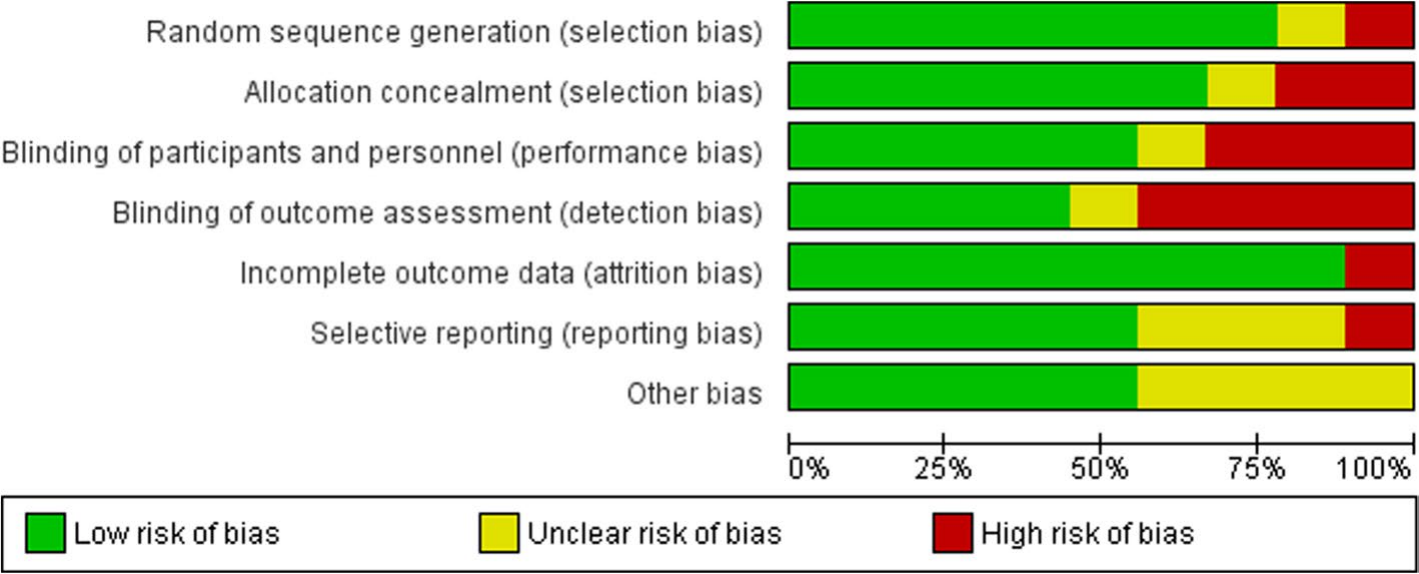
Risk of bias graph for randomized controlled trials

**Fig. 3:**
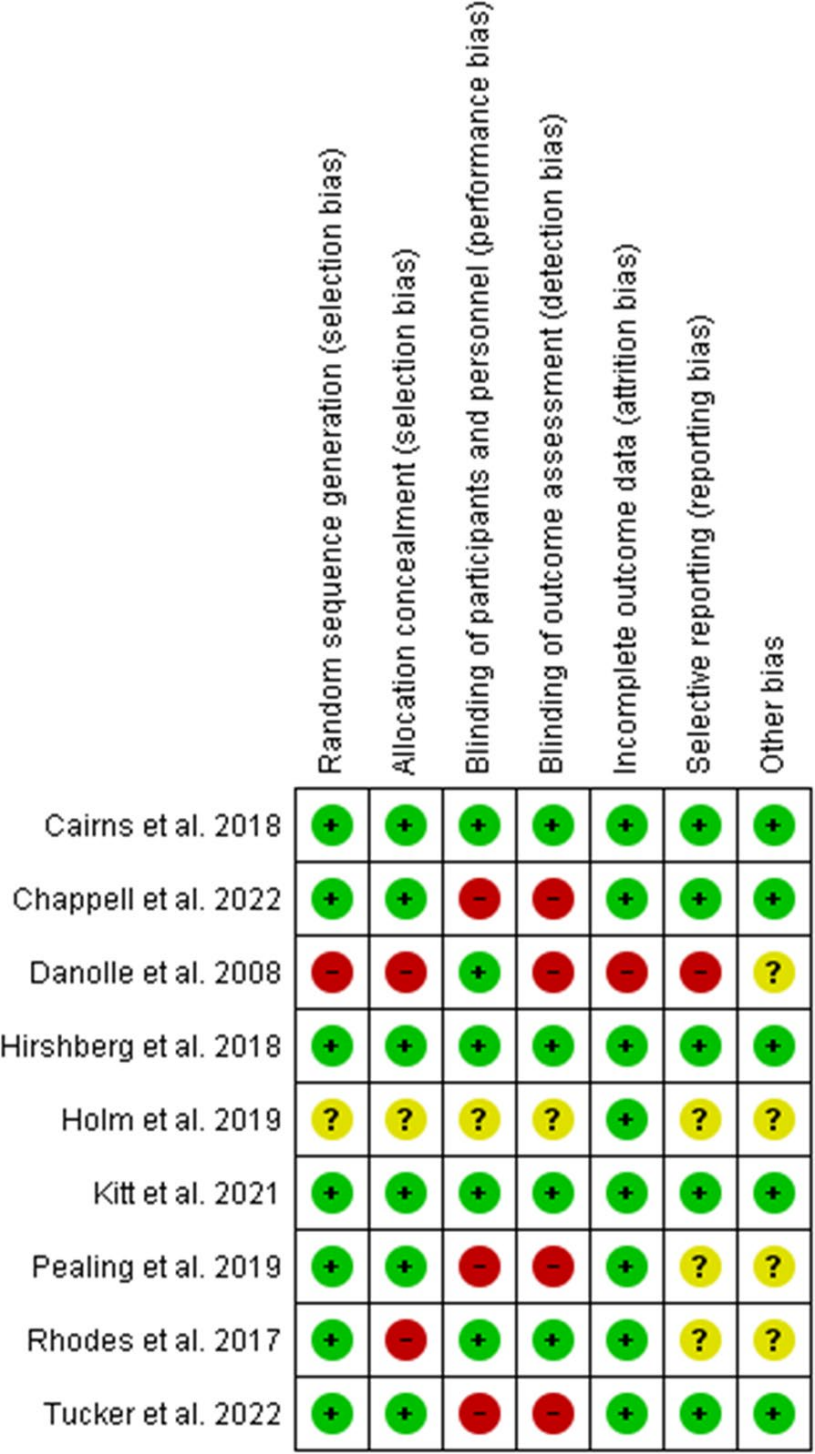
Risk of bias summary for randomized controlled trials

Overall, the included RCTs were of good quality, having a low to moderate risk of bias. Kitt et al., Hirshberg et al., and Cairns et al. were at low risk of bias in all the investigated domains of the tool [23, 27, 28]. The blinding of study participants, study personnel, and outcome detectors was not ideal in Tucker et al. and Pealing et al. [24, 25, 36], but the studies’ participants and their clinicians were required to know that they were monitored. Similarly, there was inadequate concealment of the group allocation in Rhodes et al. [34]. The risk of bias in Holms et al. could not be determined in most of the domains, and Denolle et al. were of low quality [35]. The graph and summary of these RCTs' risk of bias are provided respectively in Figs. 2 and 3.

According to NIH tools, Fukushima et al., Lanssens et al., and Kalafat et al.were of good quality, while Perry et al. and Rayburn et al. had fair quality [13, 30–33] (supplementary tables 1 and 2). Furthermore, Hoppe et al. was at low risk of bias in most of the investigated domains [29] (supplementary tables 3).

### Meta-analysis outcomes

#### Preeclampsia (%)

**Fig. 4:**
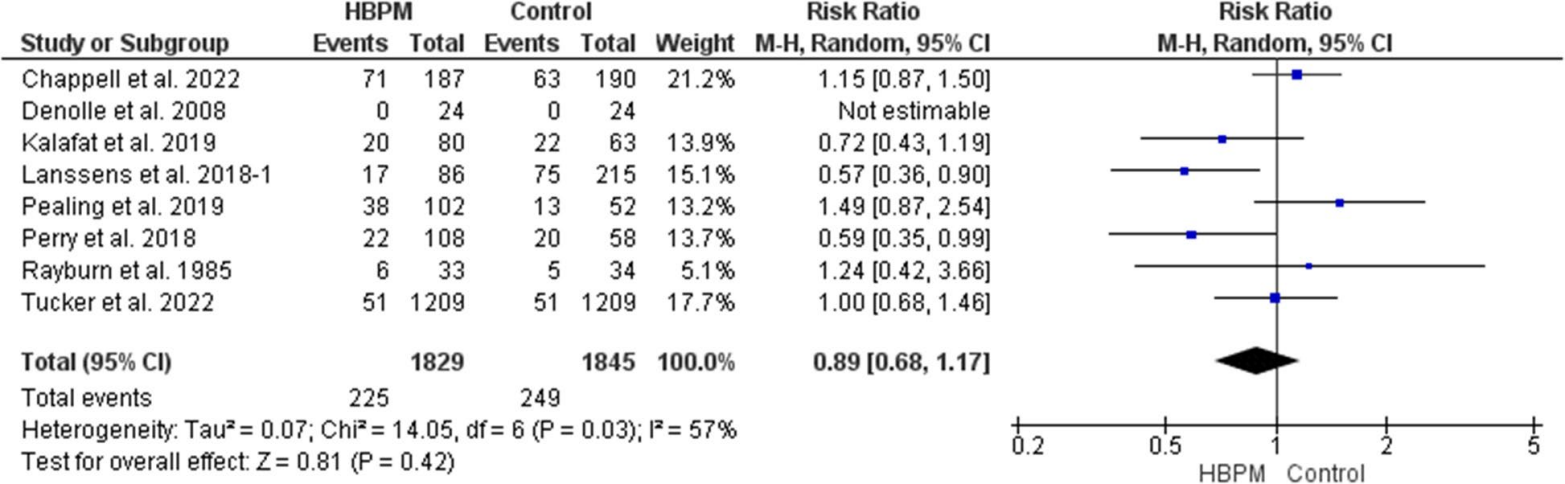
Forest plot of the analysis of preeclampsia

This meta-analysis was based upon data analyzed from eight studies, with 3674 women enrolled (1829 for HBPM and 1845 for office monitoring) [13, 24, 25, 31–33, 35, 36]. The analysis showed an insignificant variation between HBPM and office monitoring on the risk of preeclampsia (RR = 0.89; 95% CI [0.68, 1.17], *P* = 0.42), but the results showed heterogeneity across the studies (*P* = 0.03, I^2^ = 57%). And we couldn’t solve this heterogeneity (Fig. 4).

#### Induction of labor (%)

**Fig. 5:**
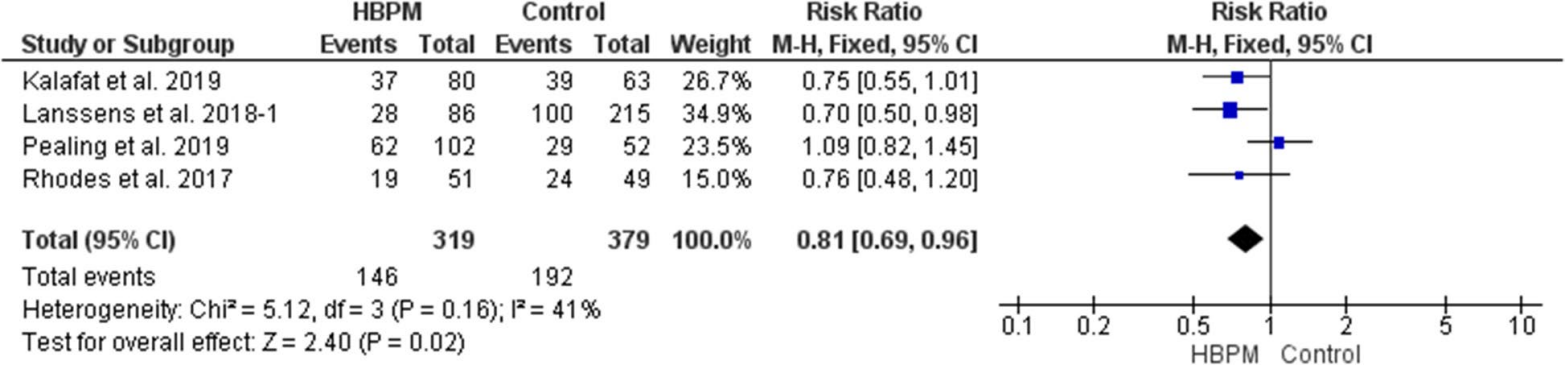
Forest plot of the analysis of induction of labor

This comparative meta-analysis was based upon data analyzed from four studies, with 698 women enrolled (319 for HBPM and 379 for office monitoring) [25, 31, 32, 34]. In comparison with office monitoring, HBPM resulted significantly in a lower risk of induction of labor (RR = 0.81; 95% CI [0.69, 0.96], *P* = 0.02), and the results showed homogeneity among the studies (*P* = 0.16, I^2^ = 41%) (Fig. 5).

#### Caesarian delivery (%)

**Fig. 6:**
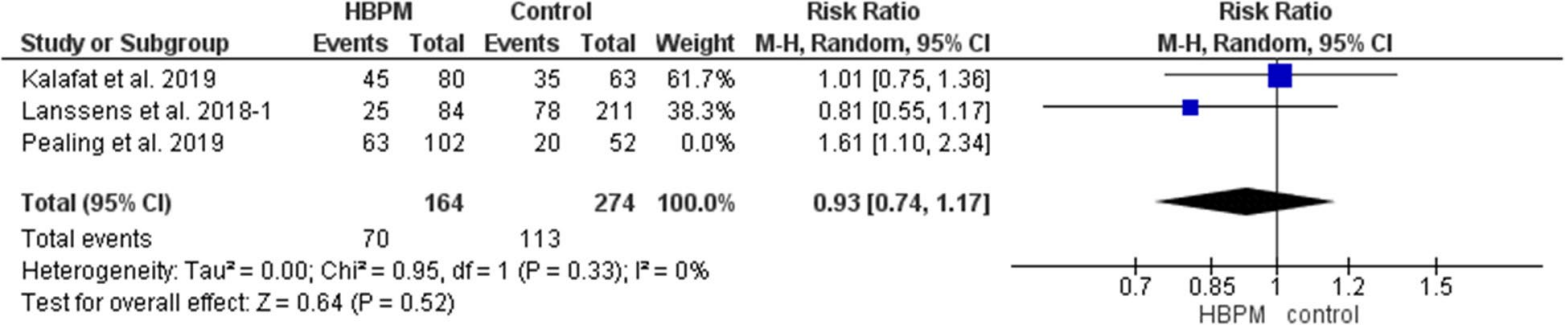
Forest plot of the analysis of caesarian delivery

This primary analysis was based on data analyzed from three studies, with 438 women enrolled (164 for HBPM and 274 for office monitoring) [25, 31, 32]. The comparative analysis revealed an insignificant variation between the HBPM and office monitoring in the risk of caesarian delivery (RR = 1.09; 95% CI [0.75, 1.58], *P* = 0.65), but the results significantly showed heterogeneity across the studies (*P* = 0.03, I^2^ = 71%). Pealing et al. [25] was excluded in a subsequent sensitivity analysis that revealed an insignificant homogeneous pooled estimate (RR = 0.93; 95% CI [0.74, 1.17], *P* = 0.52), (*P* = 0.33, I^2^ = 0%) (Fig. 6).

#### Postpartum readmission (%)

**Fig. 7:**
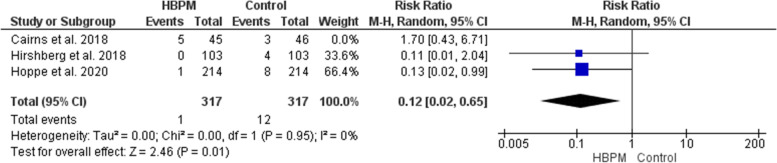
Forest plot of the analysis of postpartum readmission

This initial analysis was based upon data analyzed from three studies, with 725 women enrolled (362 for HBPM and 363 for office monitoring) [27–29]. The comparative meta-analysis showed an insignificant variation between HBPM and office monitoring in the risk of postpartum readmission (RR = 0.36; 95% CI [0.05, 2.81], *P* = 0.33). However, the results across the studies showed significant heterogeneity (*P* = 0.05, I^2^ = 67%). Cairns et al. [28] was left out in a subsequent sensitivity analysis that revealed a homogenous preference of HBPM (RR = 0.12; 95% CI [0.02, 0.65], *P* = 0.01), (*P* = 0.95, I^2^ = 0%) (Fig. 7).

#### Live birth (%)

**Fig. 8:**
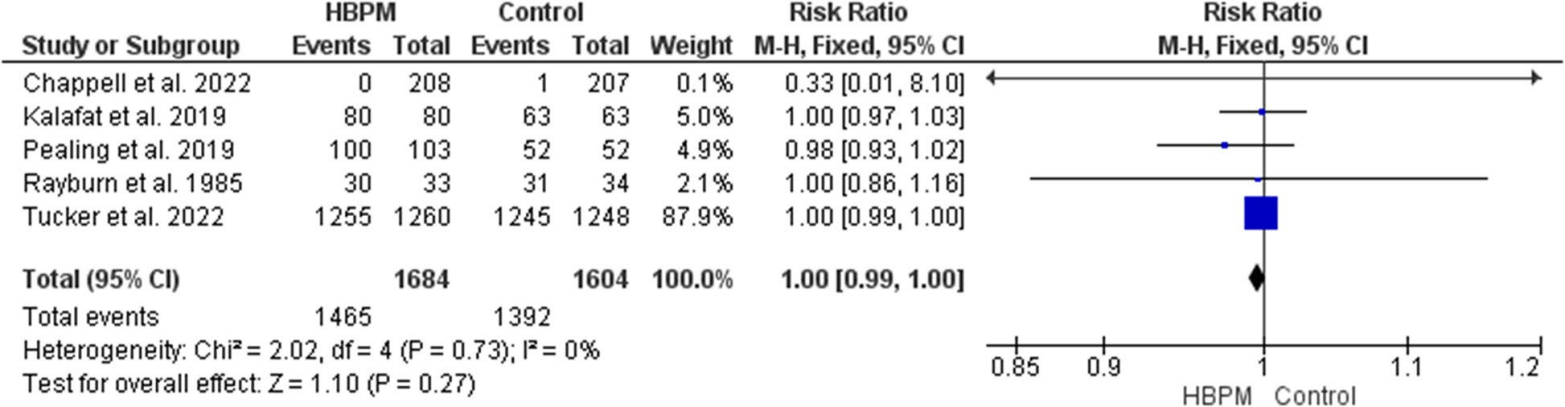
Forest plot of the analysis of live birth. Forest plot of the analysis of postpartum readmission

This analysis was based upon data analyzed from five studies, with 3288 participants enrolled (1684 for HBPM and 1604 for office monitoring) [24, 25, 31, 33, 36]. The meta-analysis of live birth revealed an insignificant variation between HBPM and office monitoring (RR = 1.00; 95% CI [0.99, 1.00], *P* = 0.36), and the results showed homogeneity among the studies (*P* = 0.73, I^2^ = 0%) (Fig. 8).

#### Gestational age at delivery (weeks)

**Fig. 9:**
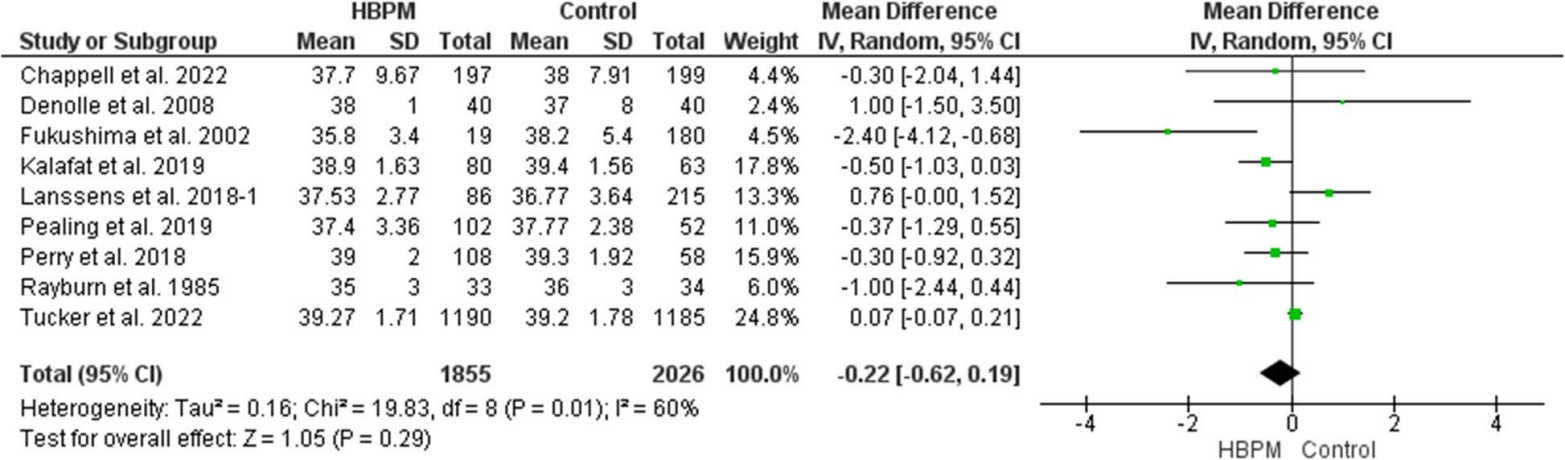
Forest plot of the analysis of gestational age at delivery

This analysis included nine studies, with 3881 participants enrolled (1855 for HBPM and 2026 for office monitoring) [13, 24, 25, 30–33, 35, 36]. No significant difference was revealed between HBPM and office monitoring in the gestational age at delivery (MD = -0.22; 95% CI [-0.62, 0.19], *P* = 0.29). However, the results across the studies showed heterogeneity (*P* = 0.01, I^2^ = 60%) with random effect. And we couldn’t solve this heterogeneity (Fig. 9).

#### Preterm delivery (%)

**Fig. 10:**
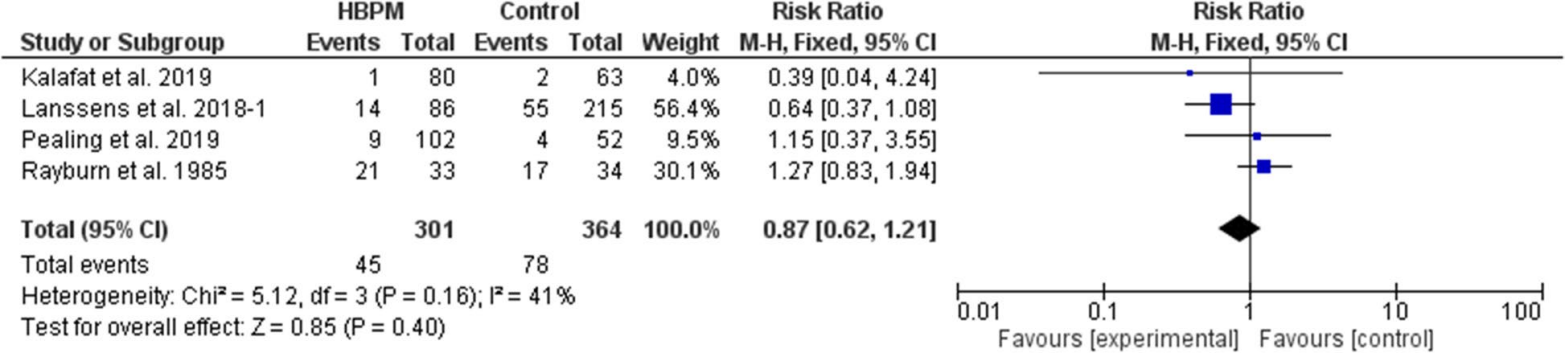
Forest plot of the analysis of preterm delivery

This comparative analysis was based upon data analyzed from four studies, with 665 participants enrolled (301 for HBPM and 364 for office monitoring) [25, 31–33]. The comparative meta-analysis showed an insignificant variation between HBPM and office monitoring in the risk of preterm delivery (RR = 0.87; 95% CI [0.62, 1.21], *P* = 0.40), and the results showed homogeneity among the studies (*P* = 0.16, I^2^ = 41%). And we couldn’t solve this heterogeneity (Fig. 10).

#### Birth weight (g)

**Fig. 11:**
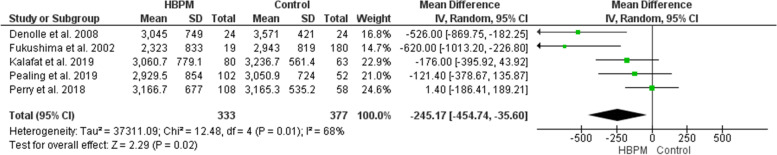
Forest plot of the analysis of birth weight

This comparative analysis was based upon data analyzed from five studies, with 710 participants enrolled (333 for HBPM and 377 for office monitoring) [13, 25, 30, 31, 35]. This analysis revealed a significant variation in the birth weight that favors HBPM over office control (MD = -245.17; 95% CI [-454.74, -35.60], *P* = 0.02). However, the results significantly showed heterogeneity across the included studies (*P* = 0.01, I^2^ = 68%). And we couldn’t solve this heterogeneity (Fig. 11).

#### Intrauterine growth restriction (%)

**Fig. 12:**
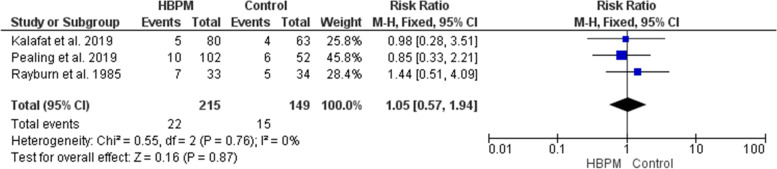
Forest plot of the analysis of intrauterine growth restriction

This analysis was based upon data analyzed from three studies, with 364 participants enrolled (215 for HBPM and 149 for office monitoring) [25, 31, 33]. The comparative analysis showed an insignificant variation between HBPM and office monitoring regarding the risk of Intrauterine growth restriction (RR = 1.05; 95% CI [0.57, 1.94], *P* = 0.87), and the results were homogenous (*P* = 0.76, I^2^ = 0%) (Fig. 12).

#### Small for gestational age (%)

**Fig. 13:**
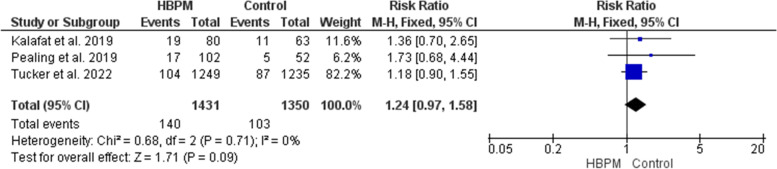
Forest plot of the analysis of small for gestational age

This analysis was based upon data analyzed from three studies, with 2781 participants enrolled (1431 for HBPM and 1350 for office monitoring) [24, 25, 31]. This analysis revealed an insignificant variation between HBPM and office monitoring in the risk of delivering a neonate that is small for gestational age (RR = 1.24; 95% CI [0.97, 1.58], *P* = 0.09), and the results showed homogeneity (*P* = 0.71, I^2^ = 0%) (Fig. 13).

#### NICU admission (%)

**Fig. 14:**
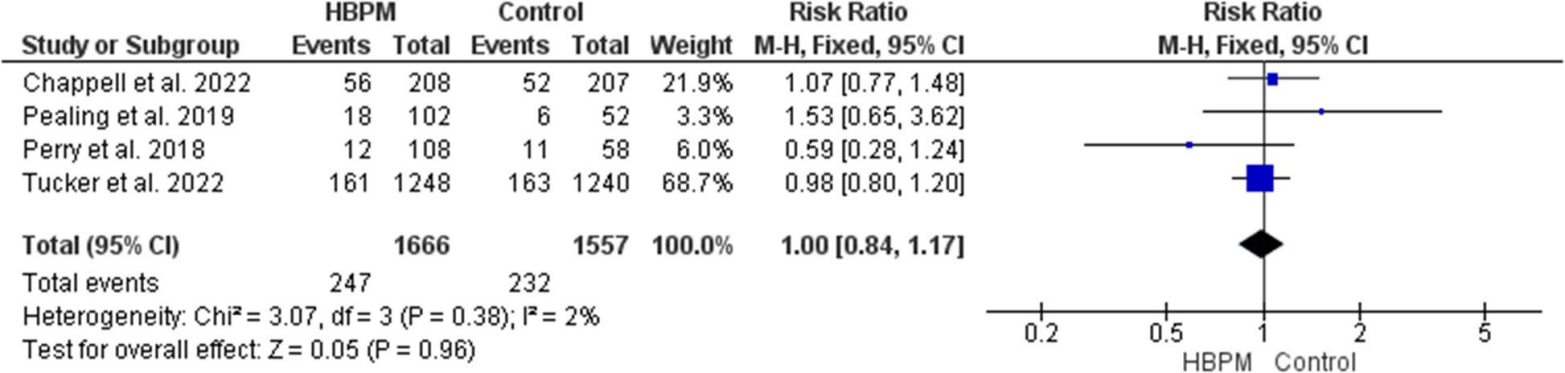
Forest plot of the analysis of neonatal intensive care unit admission

This analysis was based upon data analyzed from four studies, with 3223 participants enrolled (1666 for HBPM and 1557 for office monitoring) [13, 24, 25, 36]. The comparative meta-analysis revealed an insignificant variation between HBPM and office monitoring in the risk of NICU admission (RR = 1.00; 95% CI [0.84, 1.17], *P* = 0.96), and the results among the studies showed homogeneity (*P* = 0.38, I^2^ = 2%) (Fig. 14).

#### Adverse outcomes (%)

**Fig. 15:**
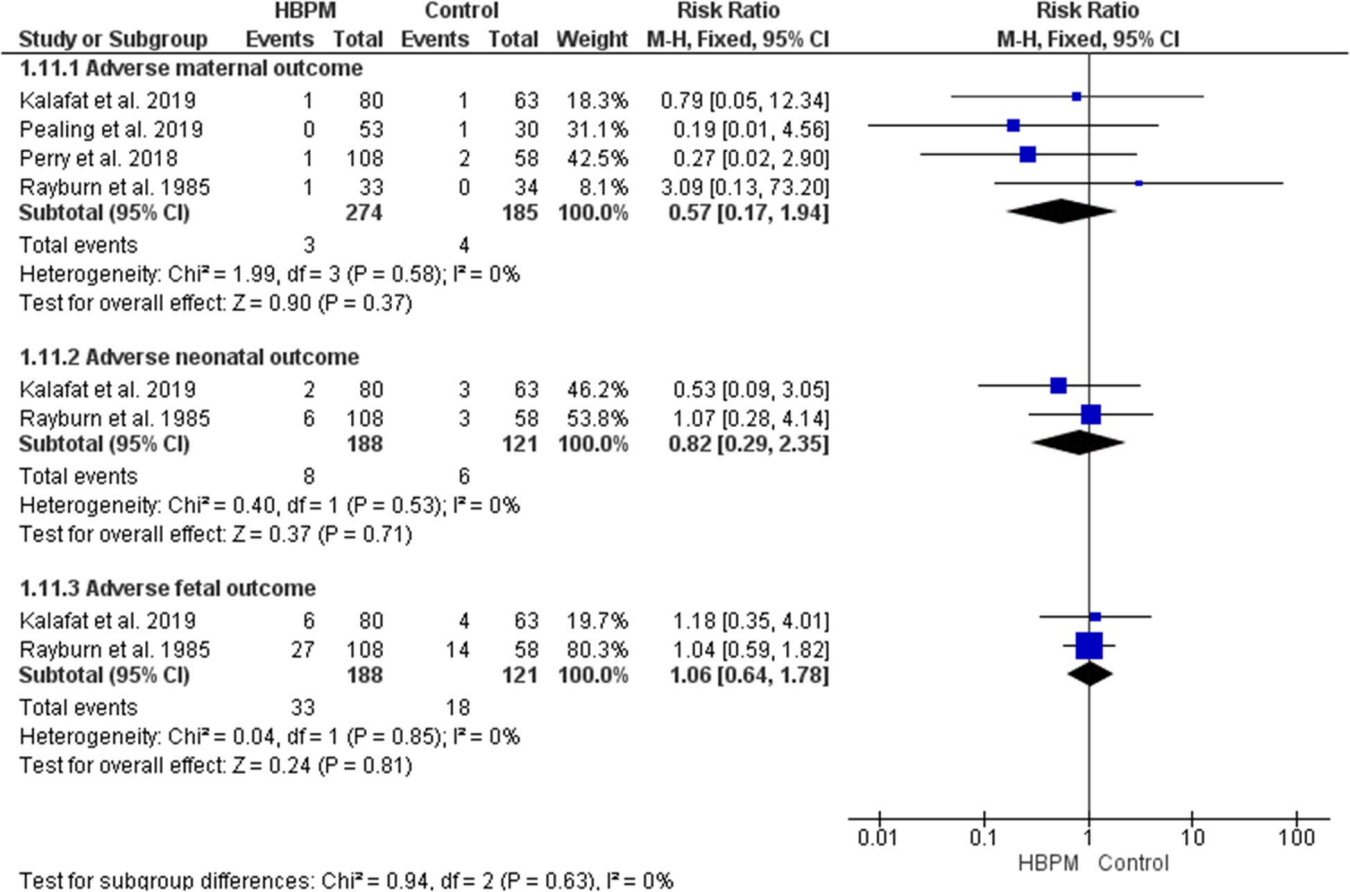
Forest plot of the analysis of adverse outcomes

A total of four studies have investigated the risk for adverse maternal, neonatal, and fetal outcomes [13, 25, 31, 33]. The comparative analyses revealed insignificant differences between HBPM and office monitoring in the risk of adverse events, and the results were homogenous among the studies (Fig. 15).

i. Adverse maternal outcomes; (RR = 0.57; 95% CI [0.17, 1.94], *P* = 0.37,) (*P* = 0.58, I^2^ = 0%).
ii. Adverse neonatal outcomes; (RR = 0.82; 95% CI [0.29, 2.35], *P* = 0.71,) (*P* = 0.53, I^2^ = 0%).
iii. Adverse fetal outcomes; (RR = 1.06; 95% CI [0.64, 1.78], *P* = 0.81,) (*P* = 0.85, I^2^ = 0%).

### Accuracy of HBPM measurements

When the measurements of mean arterial pressure from one arm at the home were compared with those acquired from the two arms in the office, no significant difference was detected (*P* = 87). This concludes that HBPM can be considered as accurate as office monitoring. However, women might get erroneous blood pressure readings in their first measurement. Several automated methods were suggested to detect and correct these possible initial erroneous measurements [26].

## Discussion

Home monitoring of blood pressure is a proposed promising alternative for office monitoring in the control of HDP. This systematic review was primarily conducted to investigate the efficacy and safety of HBPM. Fifteen studies (with a total of 5335 women enrolled) were included in our quantitative synthesis, from which, only 14 studies were eligible for quantitative synthesis. Our comparative analysis revealed a superiority of HBPM over office monitoring in regards to the risk of induction of labor, and postpartum readmission (*P* = 0.02, and 0.01 respectively). Moreover, when birth weights were compared between the two groups, a significant variation was detected in favor of HBPM (*P* = 0.02). In the analysis of other outcomes, HBPM was equally effective as office monitoring. Furthermore, maternal, neonatal, and fetal adverse outcomes were not increased with HBPM.

Our study is an update of the previous meta-analysis conducted by Kalafat et al. in 2020 [37]. Seven new studies were included in our update [23–26, 29, 34, 36], five of which were included in the quantitative analysis [24, 25, 29, 34, 36]. Our finding in reducing the risk of developing preeclampsia showed no variation between HBPM and control, and this was inconsistent with that of Kalafat et al., with the results of two new studies added to the pooled analysis [24, 36]. Results of two studies were newly included in our quantitative synthesis on the risk of induction of labor, and the pooled estimate remained consistent with that of Kalafat et al. [25, 34]. In regards to postpartum readmission, the results of a new study were included in our analysis which changes the findings from an insignificant difference in Kalafat et al. to a significant reduction of the risk with HBPM in our analysis [29]. Birth weight analysis is an analysis that was newly added in our review which showed a significant preference for HBPM over office monitoring. Several other insignificant newly added outcomes in our review were the percentage of live births and births small for gestational age in addition to the risk of caesarian delivery. With results from new studies, our review updated the analysis of weeks of gestation at delivery and the risk of preterm delivery, intrauterine growth restriction, and NICU admission. However, the pooled estimates of these outcomes remained insignificant. Similarly, new studies were included in the quantitative synthesis of the adverse maternal, neonatal, and fetal outcomes and the results remained insignificant.

Home blood pressure monitoring appears to be a promising alternative to office monitoring. This statement can be concluded from the superiority of HBPM over office monitoring in some of the investigated outcomes, in addition to equal efficacy in the remaining outcomes. None of the investigated outcomes showed the superiority of office monitoring over HBPM. HBPM resulted in fewer office visits with superior cost-saving properties. In addition to evaluating preexisting health issues, maternal age, lifestyle variables, fetal malformations, and multiple pregnancies, many other risk factors are considered while thinking about lowering clinic visits during pregnancy. The quantity and nature of antenatal care interactions a pregnant woman require to ensure a healthy pregnancy and lower the risk of perinatal fatalities can be affected by several variables. However, it’s essential to develop different modalities trying to lower the clinic visits during pregnancy, even for lower-risk pregnancies.

It was suggested previously that HBPM's first measures might be erroneous, but several automated methods were suggested as well to detect and correct these errors. Some factors that can affect the accuracy and reliability of HBPM include morning BP, evening BP, and the morning-evening difference [38]. It is also recommended to exclude the first-day home BP values as they might be erroneous [39]. There are also some automated methods for measuring blood pressure in the office setting, such as automated office BP measurement (AOBPM), which involves using a fully automated device by the patient in a quiet room separate from the office staff. However, AOBPM can also be prone to human errors if not performed correctly [40].

This alternative approach for HDP control could be considered safe also, as HBPM did not result in any significant maternal, neonatal, or fetal adverse outcomes. Our study was strengthened by the increased number of the included studies and enrolled women in comparison with the previous review. These studies were of different designs; some of the studies were observational but the majority were clinical trials that provide highly trusted evidence. Moreover, the overall quality of the eligible studies in this review was good, with a low risk of bias. In addition, our review has the advantage of studying women in both pre and postpartum periods. However, regarding our study’s limitations, the random effect model in our analysis of heterogeneous outcomes might limit the risk of getting erroneous significant results. A sensitivity analysis was conducted in some of those heterogeneous results to solve heterogeneity. We could not assess the postpartum outcomes separately due to the limited data in the included studies. Also, the issue of varied outcomes may be due to the age and different settings of the studies, monitors, and systems employed. Therefore, larger-scale multicenter RCTs are needed to resolve these limitations.

## Conclusion

Monitoring blood pressure at home is a promising alternative to office monitoring for the control of HDP. This alternative approach showed superiority in reducing the risk of induction of labor, and postpartum readmission, as well as improving birth weight. In all of the other investigated outcomes, HBPM was equally effective as office monitoring. In addition, HBPM was safe for mothers, neonates, and fetuses.

## Supplementary Information

**Additional file 1: Table S.1.** Quality assessment of Cohort studies. NR, not reported. **Table S.2.** Quality assessment of Case–control studies. NR, not reported. **Table S.3.** Quality assessment of Non-RCT. NR, not reported.

## Data Availability

All data used in this meta-analysis are included in this article and its supplementary materials, or are publicly available from the original sources.
