# Supplementary material for: An evaluation of the efficacy and the safety of home blood pressure monitoring in the control of hypertensive disorders of pregnancy in both pre and postpartum periods: a systematic review and meta-analysis
Source: BMC Pregnancy Childbirth. 2023 Aug 1;23:550. doi: 10.1186/s12884-023-05663-w (PMC10392017; doi:10.1186/s12884-023-05663-w)
Supplement: Supplementary file 1 — Additional file 1: Table S.1. Quality assessment of Cohort studies. NR, not reported. Table S.2. Quality assessment of Case–control studies. NR, not reported. Table S.3. Quality assessment of Non-RCT. NR, not reported. [file 12884_2023_5663_MOESM1_ESM.docx]

| **ID** | **NIH Quality Assessment Tool for Observational Cohort and Cross-Sectional Studies** | | | | | | | | | | | | | | | **Quality rating** | |
| --- | --- | --- | --- | --- | --- | --- | --- | --- | --- | --- | --- | --- | --- | --- | --- | --- | --- |
|  | **1. Was the research question or objective in this paper clearly stated?** | **2. Was the study population clearly specified and defined?** | **3. Was the participation rate of eligible persons at least 50%?** | **4. Were all the subjects selected or recruited from the same or similar populations (including the same time period)? Were inclusion and exclusion criteria for being in the study prespecified and applied uniformly to all participants?** | **5. Was a sample size justification, power description, or variance and effect estimates provided?** | **6. For the analyses in this paper, were the exposure(s) of interest measured prior to the outcome(s) being measured?** | **7. Was the time frame sufficient so that one could reasonably expect to see an association between exposure and outcome if it existed?** | **8. For exposures that can vary in amount or level, did the study examine different levels of exposure as related to the outcome (eg, categories of exposure or exposure measured as a continuous variable)?** | **9. Were the exposure measures (independent variables) clearly defined, valid, reliable, and implemented consistently across all study participants?** | **10. Was the exposure(s) assessed more than once over time?** | **11. Were the outcome measures (dependent variables) clearly defined, valid, reliable, and implemented consistently across all study participants?** | **12. Were the outcome assessors blinded to the exposure status of participants?** | **13. Was loss to follow-up after baseline 20% or less?** | **14. Were key potential confounding variables measured and adjusted statistically for their impact on the relationship between exposure(s) and outcome(s)?** | **total scores** |  |  |
| **Fukushima et al. 2002** | Yes | Yes | Yes | Yes | No | Yes | Yes | NA | Yes | No | Yes | No | Yes | No | 11 | Good |  |
| **Lanssens et al. 2018-1** | Yes | Yes | Yes | Yes | No | Yes | Yes | NA | Yes | Yes | Yes | NR | Yes | Yes | 11.5 | Good |  |

**Table S.1:** Quality assessment of Cohort studies. NR, not reported.

| **ID** | **NIH Quality Assessment Tool for Observational Case-control Studies** | | | | | | | | | | | | | **Quality rating** |
| --- | --- | --- | --- | --- | --- | --- | --- | --- | --- | --- | --- | --- | --- | --- |
|  | **1. Was the research question or objective in this paper clearly stated and appropriate?** | **2. Was the study population clearly specified and defined?** | **3. Did the authors include a sample size justification?** | **4. Were controls selected or recruited from the same or similar population that gave rise to the cases (including the same timeframe)?** | **5. Were the definitions, inclusion and exclusion criteria, algorithms or processes used to identify or select cases and controls valid, reliable, and implemented consistently across all study participants?** | **6. Were the cases clearly defined and differentiated from controls?** | **7. If less than 100 percent of eligible cases and/or controls were selected for the study, were the cases and/or controls randomly selected from those eligible?** | **8. Was there use of concurrent controls?** | **9. Were the investigators able to confirm that the exposure/risk occurred prior to the development of the condition or event that defined a participant as a case?** | **10. Were the measures of exposure/risk clearly defined, valid, reliable, and implemented consistently (including the same time period) across all study participants?** | **11. Were the assessors of exposure/risk blinded to the case or control status of participants?** | **12. Were key potential confounding variables measured and adjusted statistically in the analyses? If matching was used, did the investigators account for matching during study analysis?** | **Total scores** |  |
| **Kalafat et al. 2019** | Yes | Yes | No | Yes | Yes | Yes | NR | Yes | Yes | Yes | NR | Yes | 9.5 | Good |
| **Perry et al. 2018** | Yes | Yes | No | Yes | Yes | Yes | NR | Yes | Yes | Yes | NR | No | 9 | Fair |
| **Rayburn et al. 1985** | Yes | Yes | No | Yes | Yes | Yes | NR | Yes | Yes | Yes | NR | NR | 8.5 | Fair |

**Table S.2:** Quality assessment of Case-control studies. NR, not reported.

| **Observatory studies (Robans risk of bias assessment tool)** | | | | | | |
| --- | --- | --- | --- | --- | --- | --- |
| **Study ID** | **Selection of participants** | **Confounding variables** | **Measurement of exposure** | **Blinding of outcome assessment** | **Incomplete outcome data** | **Selective outcome reporting** |
| **Hoppe et al. 2020** | Low Risk | Low Risk | Low Risk | Not clear | Low Risk | Not clear |

**Table S.3:** Quality assessment of Non-RCT. NR, not reported.
